# Supplementary material for: Vaccination against COVID-19: Factors That Influence Vaccine Hesitancy among an Ethnically Diverse Community in the UK
Source: Vaccines (Basel). 2022 Jan 11;10(1):106. doi: 10.3390/vaccines10010106 (PMC8780359; doi:10.3390/vaccines10010106)
Supplement: Supplementary file 1 [file vaccines-10-00106-s001.zip › Supplementary File 1.pdf]

Table S1. Coding System– Reasons for not getting vaccinated

	**Codes**	**Definition**
**Attitudes and beliefs**		
1	Lack of trust in government	Lack of trust or mistrust. Lies. Government wants to control individuals and communities.
2	Lots of vaccinated people die	Lots of people die despite having been vaccinated.
3	Lack of trust in the vaccine	Vaccines have been rushed. They have not gone through all the trails. I don't trust it
4	Vaccines do not have 100% effectiveness	No vaccine provides total protection. Low effectiveness of some vaccines.
**Health**		
5	Sides effects of vaccines	Many people experience side effects after having the vaccine. Possible effects on fertility
6	I don't need it[1a]	I have good health, don't need it. I am not in danger. I am immune.
**Information**		
7	Lack of sufficient evidence of long-term effects of vaccines	Not enough evidence of possible harm to human health.
**Other**		
8	No clear reason	My reasons are private/personal choice. I don't want it. I need more time. I am not sure[d].
9	Health conditions[1b]	Pregnancy
10	Heard immunity[1b]	I will wait until there is herd immunity.
11	Fear of injections[1b]	I am afraid of injections/needles

[a] Modified at the end of stage 2 (as a result of discussions over discrepancies), [b] Modified at the end of stage 3.
