# Supplementary material for: Vaccination against COVID-19: Factors That Influence Vaccine Hesitancy among an Ethnically Diverse Community in the UK
Source: Vaccines (Basel). 2022 Jan 11;10(1):106. doi: 10.3390/vaccines10010106 (PMC8780359; doi:10.3390/vaccines10010106)
Supplement: Supplementary file 1 [file vaccines-10-00106-s001.zip › Supplementary File 2.pdf]

Table S2. Coding system – What would change your mind (about getting vaccinated)

	**Codes**	**Definition**
**Health**		
1	If the vaccine does not affect fertility	If vaccine does not affect fertility in young people (or others)
2	Vaccine to provide 100% protection	100% effectiveness
3	If effects of vaccine are not passed on through breast feeding	If effects of vaccine do not pass on through breast feeding or affect baby
**Information**		
4	Evidence to back up claims	Evidence or data to support claims. More research. Standard trials. FDA[1] approval.
5	Information on long-term side effects of vaccine	Evidence on long-term effects.
10	Knowing my legal rights[1a]	
**Other**		
6	Someone from my family or high profile[a] not experiencing side effects	If I witness someone in my family/close circle of friends who has had the vaccine and not experienced side effects.
7	Access to vaccines abroad	Better access to vaccines abroad (e.g., international students)
8	It depends on means of persuasion	
9	If made compulsory	If mandatory. By law. If penalties are introduced.
10	Knowing my legal rights[a]	
11	No suggestion[1 a]	

[a] Modified at the end of stage 2 (as a result of discussions over discrepancies)

[1] These were new codes added as part of the Stage 2 of analysis; [a] Modified at the end of stage 2 (as a results of discussion over discrepancies)
